# Absence of nosocomial influenza and respiratory syncytial virus infection in the coronavirus disease 2019 (COVID-19) era: Implication of universal masking in hospitals

**DOI:** 10.1017/ice.2020.425

**Published:** 2020-08-17

**Authors:** Shuk-Ching Wong, Germaine Kit-Ming Lam, Christine Ho-Yan AuYeung, Veronica Wing-Man Chan, Newton Lau-Dan Wong, Simon Yung-Chun So, Jonathan Hon-Kwan Chen, Ivan Fan-Ngai Hung, Jasper Fuk-Woo Chan, Kwok-Yung Yuen, Vincent Chi-Chung Cheng

**Affiliations:** 1 Infection Control Team, Queen Mary Hospital, Hong Kong West Cluster, Hong Kong Special Administrative Region, China; 2 Department of Microbiology, Queen Mary Hospital, Hong Kong Special Administrative Region, China; 3 Department of Medicine, Li Ka Shing Faculty of Medicine, The University of Hong Kong, Pokfulam, Hong Kong Special Administrative Region, China; 4 Department of Microbiology, Li Ka Shing Faculty of Medicine, The University of Hong Kong, Hong Kong Special Administrative Region, China

## Abstract

Universal masking for healthcare workers and patients in hospitals was adopted to combat coronavirus disease 2019 (COVID-19), with compliance rates of 100% and 75.9%, respectively. Zero rates of nosocomial influenza A, influenza B, and respiratory syncytial virus infection were achieved from February to April 2020, which was significantly lower than the corresponding months in 2017–2019.

An unprecedented outbreak of coronavirus disease 2019 (COVID-19) due to severe acute respiratory syndrome coronavirus 2 (SARS-CoV-2) continues to spread globally. The presence of presymptomatic transmission of SARS-CoV-2 led us to implement universal masking in hospitals.^1,2^ In Hong Kong, universal masking for healthcare workers (HCWs) and patients has been adopted as a part of proactive infection control measures to combat COVID-19.^3^ Because published data on the efficacy of universal masking policies to prevent nosocomial transmission of respiratory viruses are limited, we analyzed the incidence of nosocomial influenza A, influenza B, and respiratory syncytial virus (RSV) in a healthcare network in Hong Kong during the COVID-19 era compared with the historical period before universal masking was implemented (ie, the preintervention period).

## Methods

### Infection control measures for COVID-19

The study was conducted in a healthcare network comprising an acute-care, university-affiliated, teaching hospital and 4 extended-care hospitals with a total of 3,100 beds in Hong Kong. With the outbreak of COVID-19 pneumonia in Wuhan, China, our response plan changed from the alert level to the serious response level on January 4, 2020, and it further elevated to the emergency level on January 25, 2020 in Hong Kong.^3^ Universal masking was implemented for all HCWs on January 4, 2020 and enforcement began on January 25, 2020. Surgical masks were provided to all patients. In addition to the active surveillance and early isolation of suspected cases for rapid molecular diagnosis, the hospital infection control team also provided intensive training to HCWs through forums, department visits, and face-to-face training of donning and doffing of personal protection equipment.^3,4^ Hand hygiene practice was enforced.

### Compliance of infection control measures

Hand hygiene compliance was performed according the World Health Organization (WHO) protocol. Compliance with universal masking by HCWs and patients was monitored by infection control nurses (ICNs) at the bedside in wards with an open cubicle setting. The design of wards was not changed in 2020. Upon each 20-minute ward visit, ICNs also recorded episodes in which HCWs and patients wore the surgical masks improperly (defined as the mask not fully covered the nose or mouth) or did not perform hand hygiene immediately after touching the external surface of masks.

### Clinical and laboratory diagnosis of influenza and respiratory syncytial virus

In addition to the diagnosis of COVID-19, nasopharyngeal aspirates or nasopharyngeal swabs were collected from patients with fever or respiratory symptoms to rule out other respiratory viral infections, including influenza A, influenza B, and respiratory syncytial virus (RSV) using Xpert Xpress Flu/RSV (Cepheid, Sunnyvale, CA) (Supplementary File 1 online). ICNs assessed the laboratory results to identify nosocomial respiratory viral infection, which was defined as patients with onset of fever or respiratory symptoms >48 hours of hospital admission, and they advised appropriate infection control measures to prevent hospital outbreak.

We monitored the incidence of nosocomial acquisition of influenza A, influenza B, and RSV from February 2020 to April 2020, after enforcement of universal masking began on January 25, 2020. This period represents within the common seasonal influenza surge in Hong Kong, which occurs from January to April and from July to August (RSV infection occurs throughout the year in Hong Kong). The corresponding months (February–April) in 2017, 2018, and 2019 were chosen as the preintervention period for comparison.

### Statistical analysis

The χ^2^ test was used to compare independent categorical variables between groups. *P* < .05 was considered statistically significant.

## Results

### Nosocomial acquisition of influenza A, influenza B, and RSV

The number of patients tested for influenza A, influenza B, and RSV during hospitalization was comparable between the 2 periods (Table 1). Absence of nosocomial influenza A, influenza B, and RSV infection was achieved from February to April 2020 in our healthcare network. The number of nosocomial influenza A, influenza B, and RSV cases per month and per 1,000 patient days per month were significantly lower than during the preintervention period (Table 1).

**Table 1.** Nosocomial Influenza A Virus, Influenza B Virus, and Respiratory Syncytial Virus Before and During the COVID-19 Era

Variable	2017–2019 (Feb–Apr)	2020 (Feb–Apr)^a^	*P* Value
**Laboratory request for respiratory viruses detection^b^**			
Total no. of patients tested per mo, mean ± SD	1,357 ± 188	1,342 ± 355	.923
No. of patients tested per mo (NPA or NPS collected >48 h of admission), mean ± SD	110 ± 13	68 ± 16	.001
**Influenza A virus^c,d^**			
Nosocomial (NPA or NPS collected >48 h of admission)			
No. of positive per month, mean ± SD	5 ± 2.7	0 ± 0	.012
No. of positive per 1,000 patient days per month, mean ± SD	0.07 ± 0.05	0 ± 0	.023
**Influenza B virus^c,d^**			
Nosocomial (NPA or NPS collected >48 h of admission)			
No. of positive per month, mean ± SD	2 ± 3	0 ± 0	.386
No. of positive per 1,000 patient days per month, mean ± SD	0.03 ± 0.05	0 ± 0	.403
**Respiratory syncytial virus^c,d^**			
Nosocomial (NPA or NPS collected >48 h of admission)			
No. of positive per month, mean ± SD	3 ± 2	0 ± 0	.014
No. of positive per 1,000 patient days per month, mean ± SD	0.04 ± 0.02	0 ± 0	.023

Note. NPA, nasopharyngeal aspirate; NPS, nasopharyngeal swab; SD, standard deviation; HCW, healthcare worker.

a We monitored the incidence of nosocomial acquisition of influenza A, influenza B, and RSV from February 2020 to April 2020, after enforcement of universal masking in the hospitals on January 25, 2020. This period should be within the common seasonal influenza surge in Hong Kong in January–April and July–August, while RSV infection occurs throughout the year in Hong Kong.

b Laboratory request for respiratory viruses detection among HCWs who attended staff clinic for the presence of respiratory symptoms is not included in Table 1. Of 16 HCWs tested for respiratory viruses, 14 were tested in the preintervention period and 2 were tested in the study period. In the preintervention period, influenza A and influenza B were diagnosed in 4 and 3 HCWs, respectively. In the study period, no HCW was positive for influenza A, influenza B, or respiratory syncytial virus.

c The monthly breakdown number of nosocomial influenza A, influenza B, and respiratory syncytial virus per year, as well as the patient days are presented in Supplementary File 2 (online).

d No one was COVID-19 positive in the study period, Feb–Apr 2020.

### Compliance of infection control measures

The overall monthly hand hygiene compliance (mean ± SD) from February to April 2020 was 73.3 ± 2.1%, which was comparable to the corresponding figure in the preintervention period (76.9 ± 3.6%) (*P* = .14). In a 3-week audit from May 18, 2020, to June 5, 2020, 74 ward visits were made by ICNs. The compliance rates of wearing surgical masks among HCWs was 100% (889 of 889) and among adult patients was 75.9% (1,155 of 1,522). Improper wearing of surgical mask was significantly more observed among patients (132 of 1,155, 11.4%) than HCWs (11 of 889, 1.2%) (*P* < .001). In contrast, significantly more HCWs (29 of 889, 3.3%) touched the external surface of their surgical mask than patient did (18 of 1,155, 1.6%) (*P* = .01) (Table 2), but none of them performed hand hygiene immediately after touching their mask.

**Table 2.** Compliance of Wearing Surgical Masks Among Healthcare Workers and Patients

Unit	Episodes of HCWs Observed	Episodes of HCWs Wearing Surgical Masks, No. (%)	Episodes of HCWs Wearing Surgical Masks Improperly, No. (%)^a^	Episodes of HCWs Touched External Surface of Surgical Masks, No. (%)^b^	Episodes of Patients Observed	Episodes of Patients Wearing Surgical Masks, No. (%)	Episodes of Patient Wearing Surgical Masks Improperly, No. (%)^a^	Episodes of Patients Touched External Surface Of Surgical Masks, No. (%)^b^
Medicine	394	394 (100)	3/394 (0.8)	10/394 (2.5)	753	568 (75.4)	67/568 (11.8)	2/568 (0.4)
Surgery	251	251 (100)	5/251 (2.0)	12 /251 (4.8)	482	365 (75.7)	60/365 (16.4)	16/365 (4.4)
Pediatrics	128	128 (100)	2/128 (1.6)	5/128 (3.9)	NA	NA	NA	NA
Orthopedics	40	40 (100)	0 (0)	1/40 (2.5)	136	105 (77.2)	1/105 (1.0)	0 (0)
ObGyn	23	23 (100)	1/23 (4.3)	1/23 (4.3)	40	26 (65.0)	1/26 (3.8)	0 (0)
Others^c^	53	53 (100)	0 (0)	0 (0)	111	91 (82.0)	3/91 (3.3)	0 (0)
Total	889	889 (100)	11/889 (1.2)^d^	29/889 (3.3)^e^	1522	1,155 (75.9)	132/1,155 (11.4)	18/1,155 (1.6)

Note. HCWs, healthcare workers; NA, not applicable because we only observed the compliance of wearing surgical mask in adult patients; ObGyn, obstetrics and gynecology.

a Improper wearing of surgical mask is defined as that the nose or mouth is not fully covered by the surgical mask.

b None of them practice hand hygiene immediately after touching the external surface of mask.

c Including adult intensive care unit, clinical oncology, accidental and emergency unit, and mixed ward.

d 11 HCWs: 9 nurses and 2 supporting staff.

e 29 HCWs: 9 doctors, 9 nurses, 9 supporting staff, and 2 allied health staff.

## Discussion

For the infection control measures against respiratory viruses other than SARS-CoV-2, it is the general practice for our HCWs to adopt droplet precautions by wearing surgical masks within 1 m of patient contact and by practicing hand hygiene. However, lower numbers of sporadic cases of nosocomial influenza A, influenza B, and RSV were observed in the preintervention period despite of our infection control practice. In the COVID-19 era, HCWs and patients were additionally required to wear surgical masks at all times in hospitals. Although the hand hygiene compliance of HCWs was comparable before and during the COVID-19 era, the policy of universal masking may be an important contributing factor in achieving zero nosocomial infections of influenza A, influenza B, and RSV.

Universal masking may reduce the shedding of SARS-CoV-2, or other respiratory viruses, from symptomatic and asymptomatic persons and thus reduce the environmental contamination, as illustrated in our recent study.^5^ In addition, wearing surgical masks may also prevent the maneuvers of nose picking and eyes touching, a subconscious behavior^6^ that poses a risk of self-inoculation of pathogens from the environment via the contaminated hands. This factor is the reason we have highly promoted our institutionally designed sixth moment, “hand hygiene before touching your mucous membrane,” together with the practice of WHO Five Moments for Hand Hygiene.^7,8^

Compliance with universal masking was monitored between May and June 2020, 4 months after the activation of emergency response level when universal masking was implemented in our hospitals. Compliance with universal masking among HCWs remains 100%, suggesting that the practice of universal masking among HCWs is sustainable in the COVID-19 era. However, a few HCWs wore surgical masks improperly and did not practice hand hygiene immediately after touching the external surface of masks, which indicates the need for further education. Understandably, not all audited patients wore surgical masks because of their unconsciousness or unstable clinical conditions. However, wearing of surgical masks by either HCWs or patients in hospital successfully prevented nosocomial influenza A outbreak in the 2009 pandemic.^9^ In our hamster model for COVID-19, surgical mask partition placed between cages housing SARS-CoV-2-infected hamsters and cages housing exposed naive hamsters significantly reduced the rate of non-contact transmission.^10^

Universal masking in hospitals in the COVID-19 era deserves further investigation. Given the others enhanced infection control measures for COVID-19 pandemic in 2020 as potential cofounding variables, universal masking appears to be a key measure to control the transmission of respiratory viruses, as well as achieving zero nosocomial transmission of COVID-19, influenza A, influenza B, and RSV in our healthcare network.

## Supplementary material

For supplementary material accompanying this paper visit http://dx.doi.org/10.1017/ice.2020.425. click here to view supplementary material
