# Supplementary material for: Absence of nosocomial influenza and respiratory syncytial virus infection in the coronavirus disease 2019 (COVID-19) era: Implication of universal masking in hospitals
Source: Infect Control Hosp Epidemiol. 2020 Aug 17:1–4. doi: 10.1017/ice.2020.425 (PMC7468684; doi:10.1017/ice.2020.425)
Supplement: Supplementary file 1 [file icesup.zip › S0899823X20004250sup001.docx]

Supplementary file 1.

The nasopharyngeal aspirates (NPA) or nasopharyngeal swabs (NPS) are submerged into the viral transport medium (VTM) and delivered to the microbiology laboratory within 4 hours. In June 2016, we introduced the Xpert Xpress Flu/RSV (Cepheid, Sunnyvale, CA) for rapid detection of influenza A, influenza B, and respiratory syncytial virus (RSV), in parallel with and gradually replacing the conventional immunofluorescence viral antigen detection test. The NPA or NPS were tested by Xpert Xpress Flu/RSV as previously described.^1^ Briefly, 300 μL of each specimen in VTM was directly loaded into the Xpert cartridge. The cartridge was loaded into the GeneXpert XVI system (Cepheid, Sunnyvale, CA). Results were available within 40 minutes.

Reference:

1. Chen JH, Lam HY, Yip CC, Cheng VC, Chan JF, Leung TH, et al. Evaluation of the molecular Xpert Xpress Flu/RSV assay vs. Alere i Influenza A & B assay for rapid detection of influenza viruses. Diagn Microbiol Infect Dis. 2018;90:177-180.
